# Supplementary material for: Alzheimer’s Aβ catalyzes Tau phase separation and aggregation via early nanocluster solubilization
Source: Nat Commun. 2026 Mar 10;17:3755. doi: 10.1038/s41467-026-70083-1 (PMC13106686; doi:10.1038/s41467-026-70083-1)
Supplement: Supplementary file 1 — Supplementary Information [file 41467_2026_70083_MOESM1_ESM.pdf]

Alzheimer's Aβ Catalyzes Tau Phase Separation and Aggregation via Early Nanocluster Solubilization

Xun Sun[1,#], Yiming Tang[2,3,#], Xue Wang[1,#], Guadalupe Pereira Curia[1], Rebecca Sternke-Hoffmann[1], Cecilia Mörman[1,4], Juan Atilio Gerez[5], Roland Riek[5], Guanghong Wei[2,3,*], Jinghui Luo[1,*]

1. Center for Life Sciences, Paul Scherrer Institute, Villigen PSI, Switzerland

2. Key Laboratory for Computational Physical Sciences of Ministry of Education, Fudan University, Shanghai, PR China.

3. State Key Laboratory of Surface Physics, Department of Physics, Fudan University, Shanghai, PR China.

4. Department of Medicine Huddinge, Karolinska Institutet, Huddinge, Sweden

5. Institute of Molecular Physical Science, Department of Chemistry and Applied Biosciences, ETH Zurich, Zurich, Switzerland

#. Contributed equally

* Corresponding authors. E-mail address: ghwei@fudan.edu.cn, jinghui.luo@psi.ch

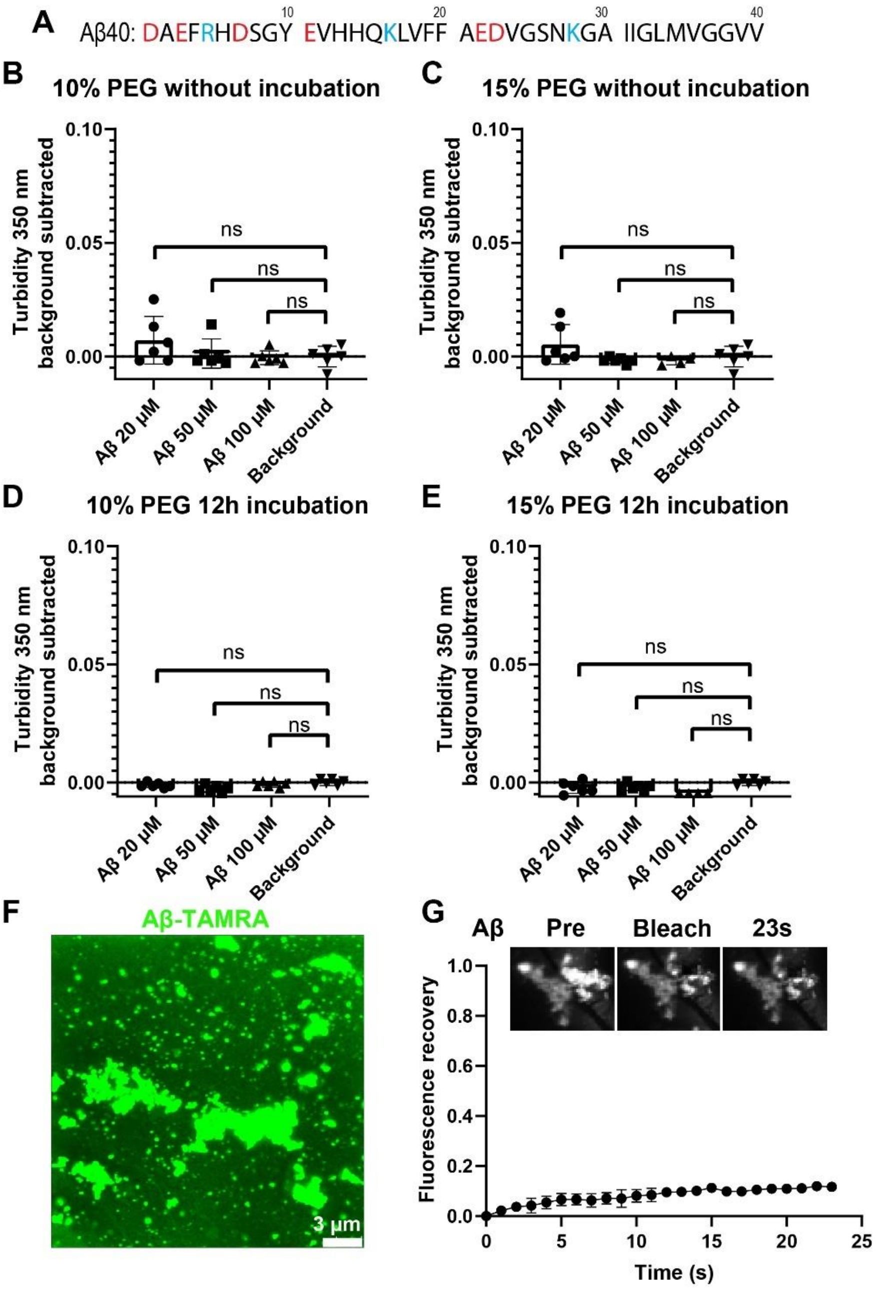

Supplementary Figure 1. Aβ40 cannot undergo LLPS in *vitro*.

A, The sequence of Aβ40. The negatively charged residues (Asp + Glu) is marked in red. The positively charged residues (Arg + Lys) is marked in blue.

B-E, Turbidity measurements of Aβ40 at different concentration and different PEG-8000 percentage with or without incubation. 20, 50, 100 μM of Aβ40 in the presence of 10% PEG without (**B**) or with (**D**) 12h incubation or in the presence of 15% PEG without (**C**) or with (**E**) 12h incubation at room temperature. The data represent the mean ± SEM. (n=6).

F, Representative fluorescence images of Aβ40 (TAMRA labelled) in the presence of 10% of PEG, reveals that Aβ40 forms aggregates not droplets.

G, Representative FRAP measurements of Aβ40 reveals solid like property of Aβ40 aggregates. The data represent the mean ± SEM. (n=3).

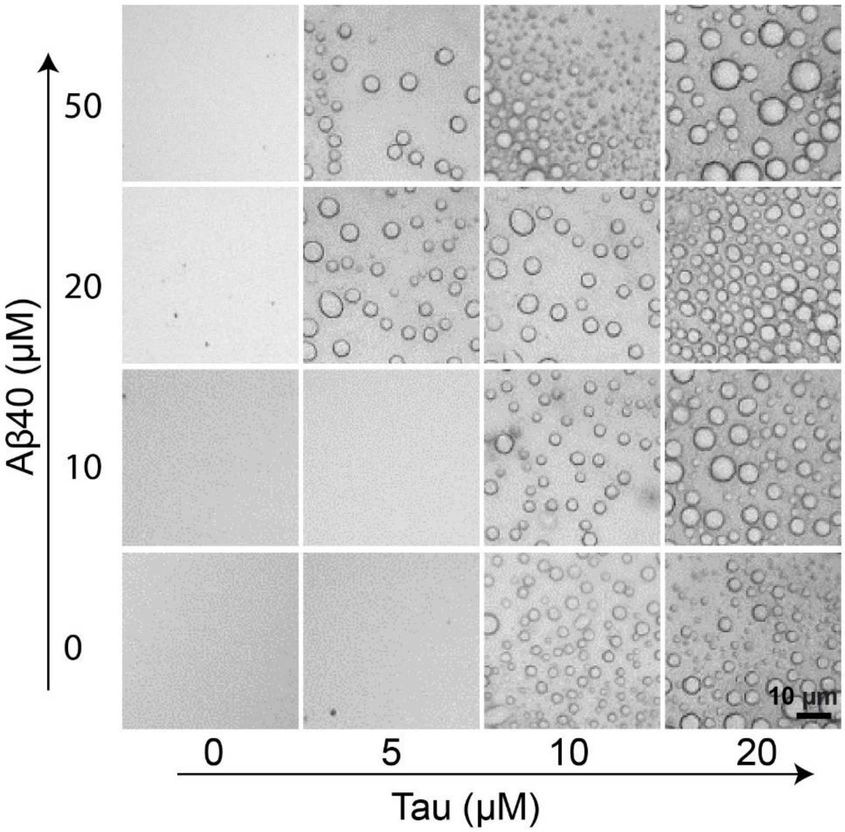

Supplementary Figure 2. Representative images corresponding to diagram (Fig. 1B) of phase separated droplets in the presence of various concentrations of Tau and Aβ40 showing the regime of Tau LLPS in the presence of 10% PEG. Scale bar in the images is 10 μm**.**

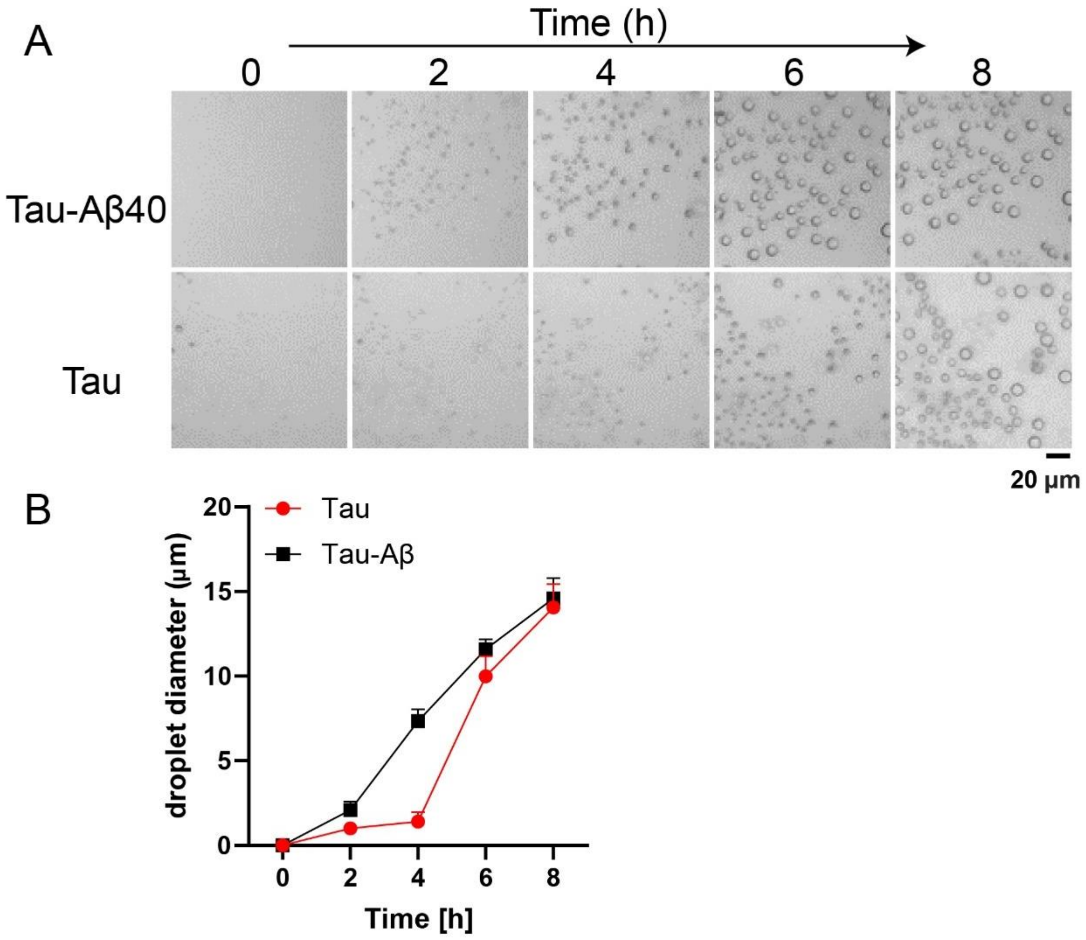

Supplementary Figure 3. Aβ40 accelerates Tau LLPS *in vitro*.

A, Representative time lapse images of phase separated droplets of Tau with or without Aβ40 in the presence of 10% PEG. Scale bar in the images is 20 μm.

B, Quantification of the diameter of droplets corresponding to A. The total number of droplets accounted is n=15 from 3 independent microscopy images.

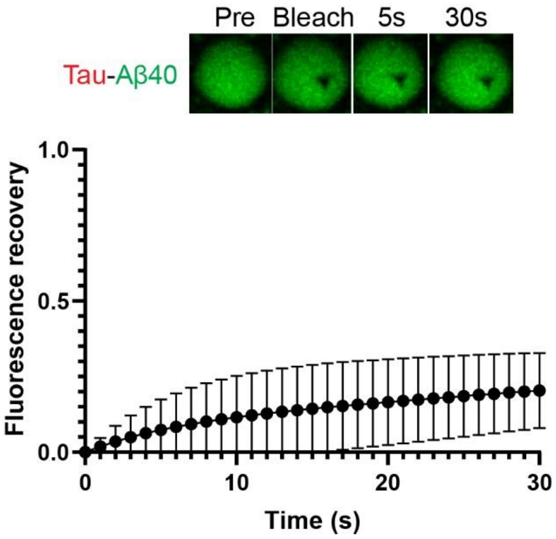

Supplementary Figure 4. Representative FRAP images (up) and analysis (bottom) of 10 μM Tau droplet in the presence of 10 μM Aβ40. The data represent the mean ± SEM. (n=4). Buffer condition: 50 mM PB, 150 mM NaCl (pH 7.4), 10% PEG8000.

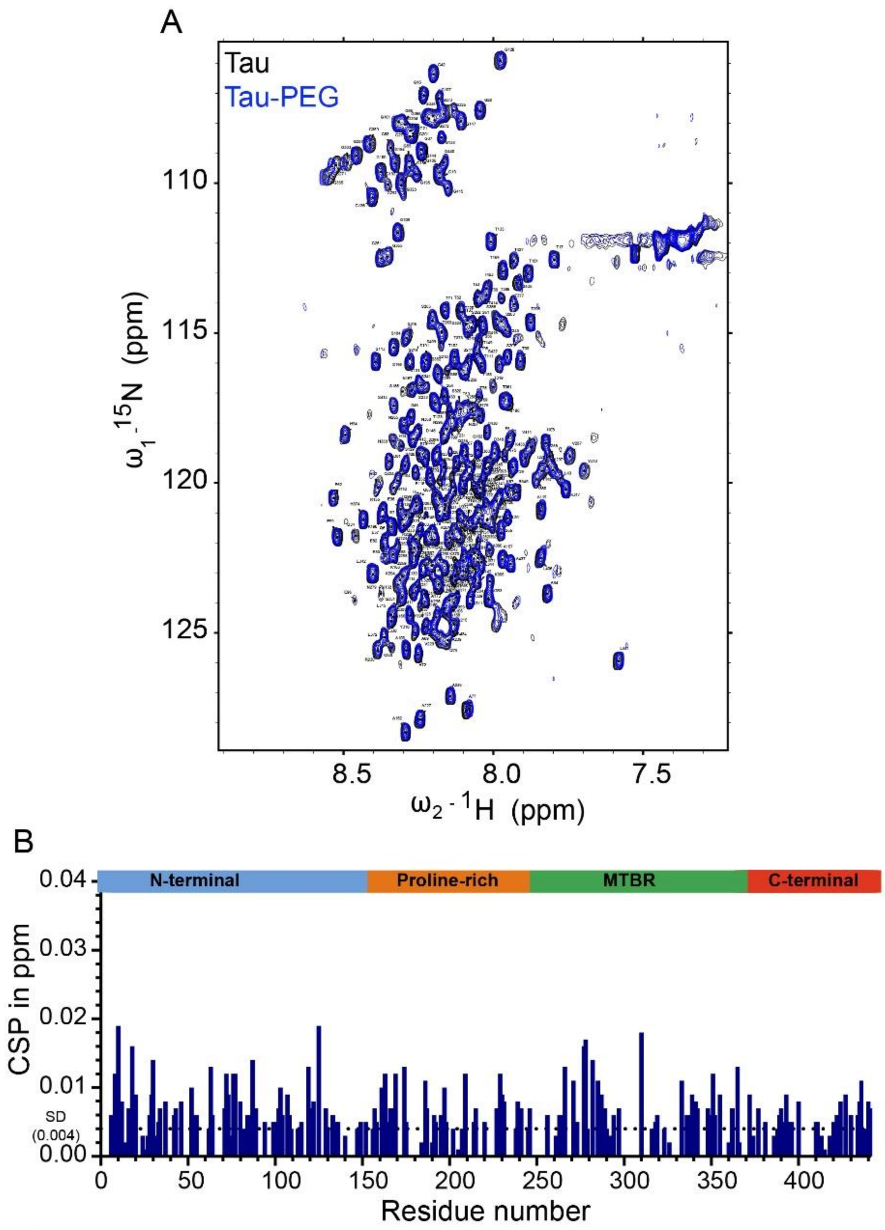

Supplementary Figure 5. 2D NMR ^{1}H-^{15}N HSQC spectrum of monomeric Tau in the absence and presence of 10% PEG8000.

A-B: 2D NMR ^{1}H-^{15}N HSQC experiments with 100 µM ^{15}N-labeled monomeric Tau in the absence (black) and presence of 10% PEG8000 (blue). The chemical shift perturbations (**B**). The experiments were performed in 20 mM sodium phosphate (pH 6.3) buffer.

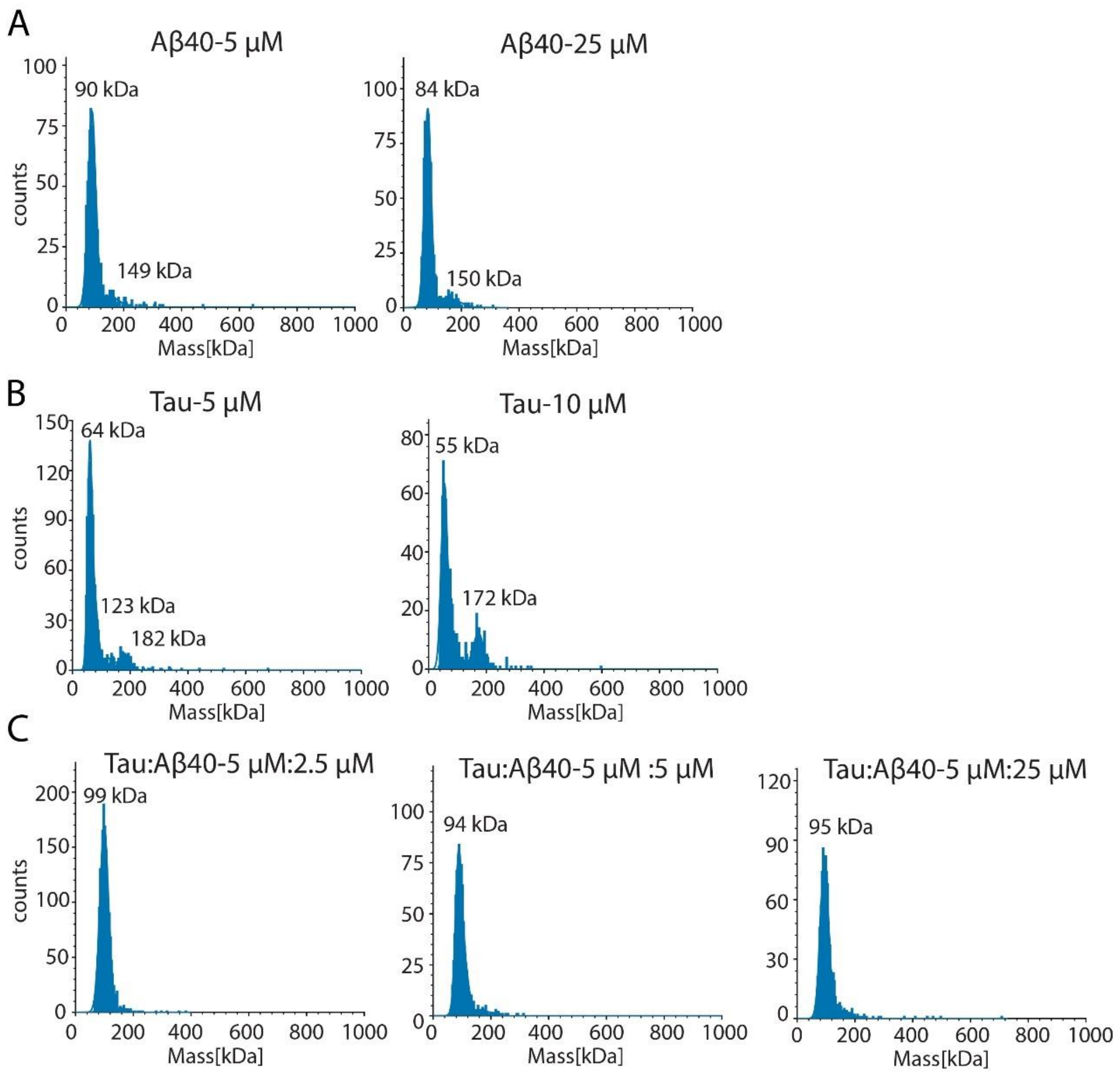

Supplementary Figure 6. Aβ40 dissolves Tau nanocluster in mass photometry.
A-C, Mass photometry of varied concentrations of Aβ40 (**A**), Tau (**B**) and Tau-Aβ40 mixture (**C**) at different ratios. MW histograms and Gaussian fitting were obtained from mass photometry measurements in a 50 mM PB, 150 mM NaCl buffer (pH 7.4).

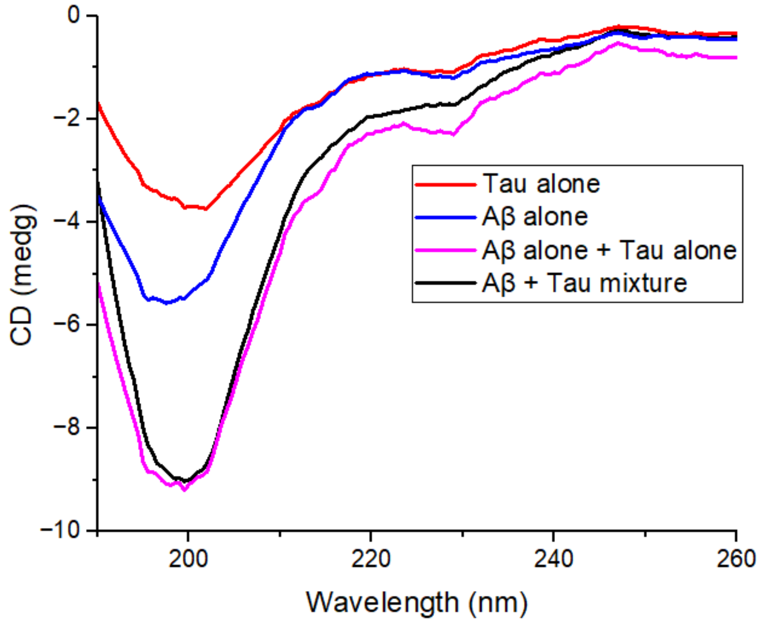

Supplementary Figure 7. CD spectroscopy measurements of Tau, Aβ, and their mixture. Far-UV CD spectra comparing 10 μM Tau alone (red), 10 μM Aβ alone (blue), Tau alone + Aβ alone signal (magenta) and the Tau-Aβ mixture (black). CD measurements were performed in the far-UV region (190-260 nm) and data are averaged from 3 triplicates and expressed as circular dichroism signal (mdeg). Buffer condition: 50 mM PB, 150 mM NaCl, pH 7.4.

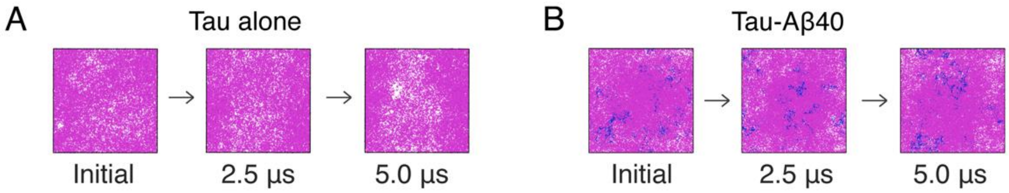

Supplementary Figure 8. Snapshots at three representative time points from simulations of a preformed Tau condensate (A) and a Tau-Aβ40 condensate (B) in a cubic simulation box.

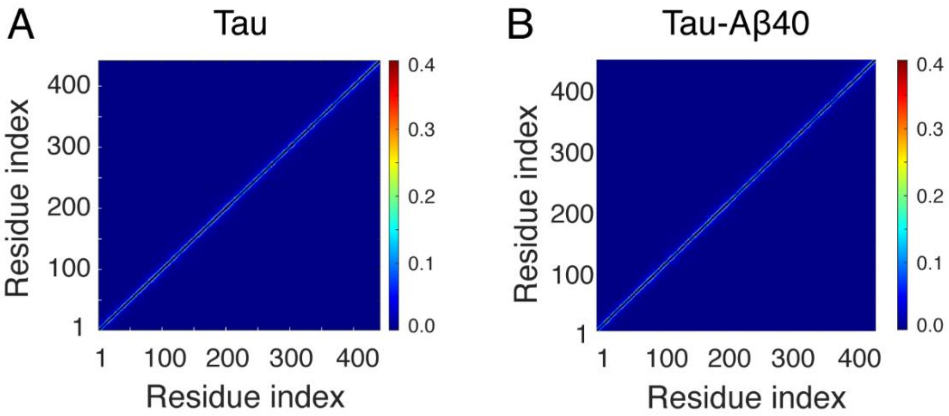

Supplementary Figure 9. Intra-molecular contact numbers between each pair of Tau residues in the (A) Tau alone system and (B) Tau-Aβ40 system.

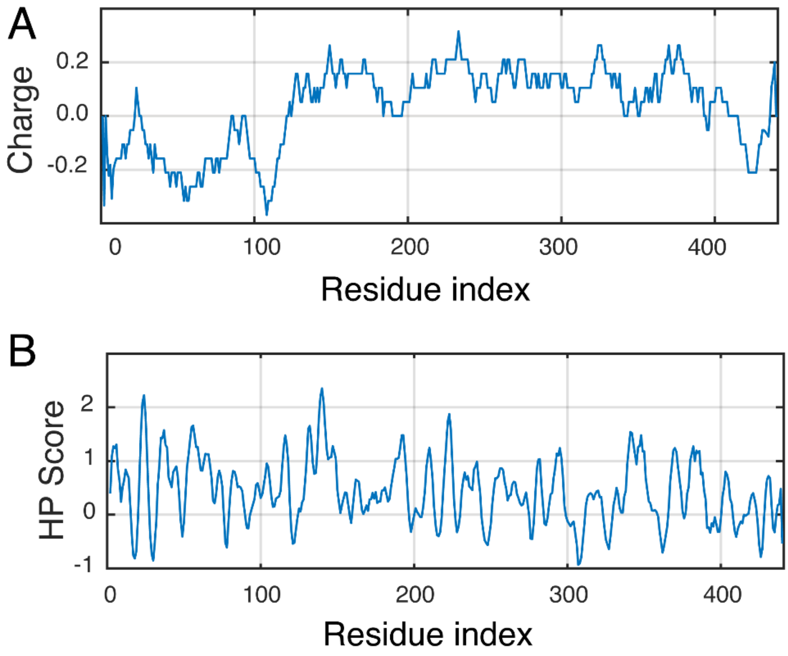

Supplementary Figure 10. Charge density (A) and hydrophobicity (B) score of each Tau residue.

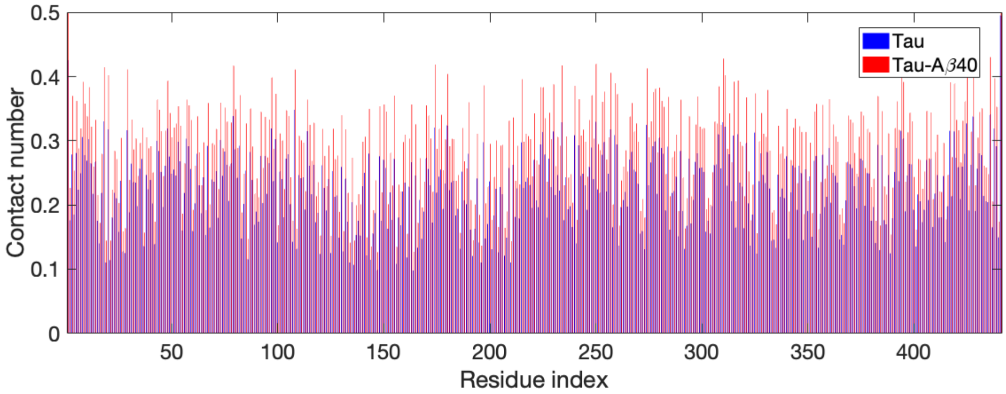

Supplementary Figure 11. Accumulated inter-molecular contact number of each Tau residue in the Tau system and in the Tau-Aβ40 system.

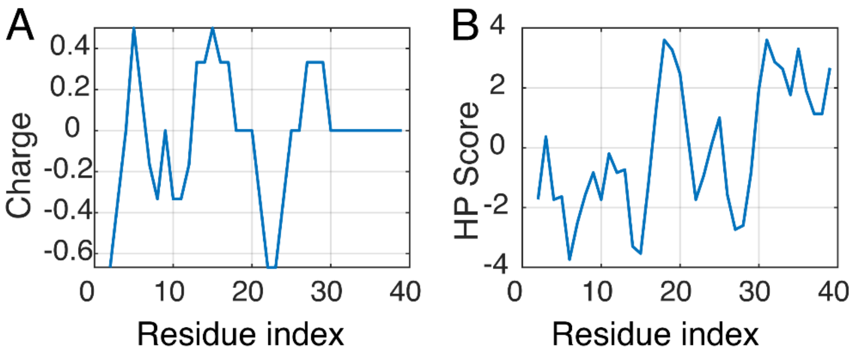

Supplementary Figure 12. Charge density and hydrophobicity score of each Aβ40 residue.

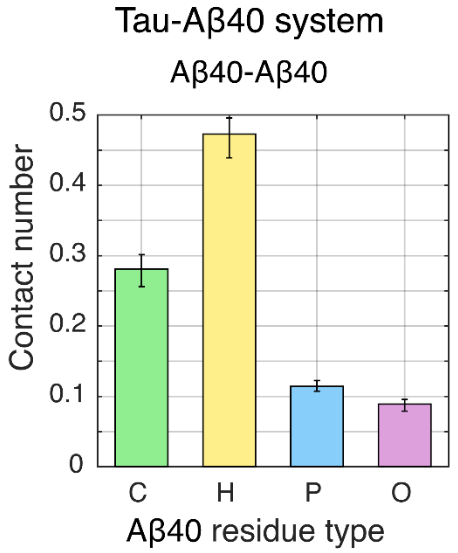

Supplementary Figure 13. Accumulated inter-Aβ40 contact numbers of each pair of Aβ40 residue types in the Tau-Aβ40 system.

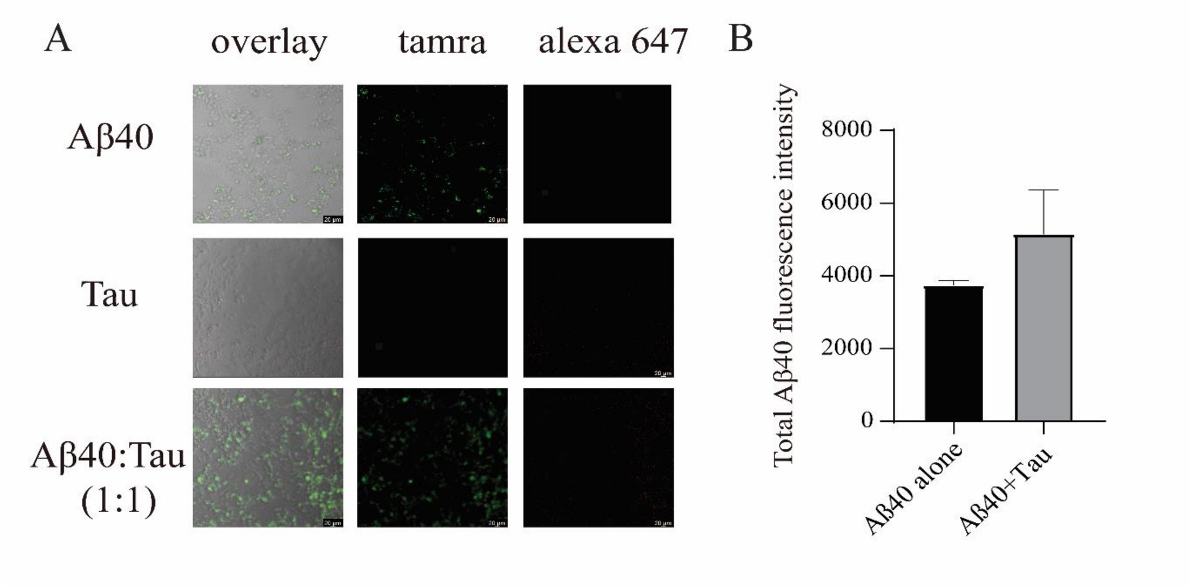

Supplementary Figure 14. (A) Representative confocal images of SH-SY5Y cells after 24 h incubation with 15 μM Aβ40, 15 μM Tau, or a mixture of 15 μM Aβ40 and 15 μM Tau. Aβ40 was detected in the TAMRA channel (shown in green) and Tau in the Alexa 647 channel (shown in red). Left column: transmitted-light/fluorescence overlay; middle: TAMRA channel; right: Alexa 647 channel. Scale bar, 20 μm. (B) Quantification of total Aβ40 fluorescence intensity in the Aβ40 alone and Aβ40+Tau groups shown in (A).

Supplementary Table 1. Fitting of density profiles to critical function $\boldsymbol{y(t) = (\Delta\rho)^{1/\beta} = kT - kT_c}$. The confidence bounds of each parameter are provided in the brackets.

System	k ($\times 10^6$ mg/mL/K)	T_c (K)
Tau alone	5.98 (5.93, 6.04)	347.30 (347.26, 347.35)
Tau-Aβ40	5.85 (5.68, 5.99)	351.50 (351.31, 351.86)
